# Decreased Peripheral Natural Killer Cells Activity in the Immune Activated Stage of Chronic Hepatitis B

**DOI:** 10.1371/journal.pone.0086927

**Published:** 2014-02-10

**Authors:** Yuan Li, Jiu-Jun Wang, Shan Gao, Qian Liu, Jia Bai, Xue-Qi Zhao, You-Hua Hao, Hong-Hui Ding, Fan Zhu, Dong-Liang Yang, Xi-Ping Zhao

**Affiliations:** 1 Department of Infectious Diseases, Tongji Hospital, Tongji Medical College, Huazhong University of Science and Technology, Wuhan, P. R. China; 2 Division of Clinical Immunology, Tongji Hospital, Tongji Medical College, Huazhong University of Science and Technology, Wuhan, P. R. China; Karolinska Institutet, Sweden

## Abstract

**Background & Aims:**

The natural course of chronic hepatitis B virus (HBV) infection is characterized by different immune responses, ranging from immune tolerant (IT) to immune activated (IA) stages. In our study, we investigated the natural killer (NK) cells activity in patients at different immunological stages of chronic HBV infection.

**Methods:**

Blood samples obtained from 57 HBeAg positive patients with chronic hepatitis B (CHB), including 15 patients in the immune tolerant (IT) stage, 42 patients in the immune activated (IA) stage, and 18 healthy individuals (HI). The analyses included flow cytometry to detect NK cells, the determination of cytokine levels as well as of surface receptor expression and cytotoxicity.

**Results:**

NK cells in peripheral blood were significantly lower in patients in the IA stage of CHB compared to HI (p<0.05). Patients in the IA stage of CHB had lower levels of NK cells activating receptor NKp30 and NKG2D expression, cytokine interferon-γ (IFN-γ) and tumor necrosis factor-α (TNF-α) production, as compared to patients in the IT stage and HI, respectively (p<0.05). Cytotoxicity of NK cells was lower in patients in the IA stage of CHB compared to patients in the IT stage and HI, respectively (p<0.05). The level of IFN-γ but not level of TNF-α and cytotoxicity of NK cells was inversely correlated with serum HBV load in patients with CHB. Peripheral NK cells activity did not correlate with ALT level.

**Conclusion:**

NK cells activity was lower in CHB patients, especially in those in the IA stage.

## Introduction

Hepatitis B virus (HBV) infection is a leading cause of liver diseases worldwide, especially in Asia and is associated with a wide spectrum of clinical manifestations, ranging from an asymptomatic course to active hepatitis B with progression to liver cirrhosis and hepatocellular carcinoma (HCC).

While HBV is not directly a cytopathic, the pathogenesis of HBV-related liver disease is immune mediated. The natural course of chronic HBV infection can present as different immunological stages, including immune tolerance and immune activation. The different immunological states of chronic HBV infection are associated with different levels of viral replication and inflammation as well as cellular immunity and humoral immunity [1]. The immune tolerant (IT) stage is characterized by HBeAg positivity, high levels of serum HBV DNA, normal serum alanine aminotransferase (ALT) levels and normal or minimally abnormal liver histology [2], [3]. The HBV-specific immune response in the IT stage is very low or even absent. The immune activated (IA) stage is characterized by low levels of serum HBV DNA, elevated ALT levels, and active inflammation and even fibrosis in liver tissue [2], [3]. In the IA stage, there is an HBV-specific immune response that is not strong enough, however, to result in HBV elimination.

Recently, the pathogenesis of innate immunity, including NK cell activity has been stressed in patients with viral hepatitis [4]–[7]. NK cells, which enriched markedly in liver and account for around one-third of total intrahepatic lymphocytes, are an important part of the innate immune system [8]. NK cells can not only directly kill virus-infected target cells without antigen-specific priming, but also regulate the adaptive immune response by producing cytokines such as IFN-γ and TNF-α, and thus take a central role in infection control [8]. Cross-talk of NK cells with CD8+ T cells strongly impact on the outcome of HBV infection. Early large amount of IFN-γ production by NK cells contribute to the initial control of infection and to allow timely development of an efficient adaptive immune response in self-limited acute HBV infection [9]–[11]. However, NK cells can negatively regulate specific antiviral immunity in chronic hepatitis B by directly killing HBV-specific CD8+ T cells which expressed TRAIL receptor, taking an important part in the failure of HBV elimination [12]. Besides, NK cells have important role in the pathogenesis of liver damage and inflammation through TRAIL- and Fas-mediated death [13], [14]. The intensity and quality of NK cells function is determined by the precisely and dynamic coordinated balance of activating and inhibitory signals through their array receptors [15], [16]. The activating receptors include NKp30, NKp46, NKG2C and NKG2D. NKp30 and NKp46 recognize MHC-independent ligands and transmit activating signals and induce NK cell cytotoxicity [15], while NKG2C and NKG2D recognize MHC-class I molecules. NKG2A, an inhibitory NK cells receptor, recognizes MHC-class I like molecules and transmits negative signals. NK cells activity is also regulated by cytokines microenvironment, with which the major immunosuppressive cytokines are TGF-β and IL-10 [17], [18]. It had been reported that the frequency, subsets, surface receptors as well as the activity of NK cells had changed in patients infected with HBV as well as hepatitis C virus (HCV) [17], [19]–[24], and the activity of NK ells altered after anti-viral treatment [25].

In this study, we analyzed NK cells activity in different immunological stages of chronic HBV infection. We found that NK cells activity is decreased in patients with chronic HBV infection, especially in patients in the IA stage. Unlike previous reports that NK cells function had dichotomy change in cytotoxicity and cytokine secretion [23], [26], [27], our study shown that both the cytotoxicity and secretion of cytokines were decreased, along with the decreased expression of activating receptors of NKp30 and NKG2D in NK cells in patients with chronic hepatitis B.

## Results

### PBMC subsets in patients with chronic HBV infection

CD3^−^CD56^+^ NK cells, CD4^+^ T cells and CD8^+^ T cells have all been shown to be involved in the antiviral immune response [30], [31]. The frequency of these effector cells in patients with chronic HBV infection is summarized in Table 1. In patients with CHB in the IA stage, the frequency of NK cells was lower than that in HI (p<0.05). Non difference of NK cells frequency was observed between patients in the IA and IT stage, respectively. There were no obvious differences in the frequency of CD56dim and CD56bright NK cells between patients groups (data not shown). With respect to the frequency CD4^+^ T cells and CD8^+^ T cells there was no significant difference between the three study groups.

**Table 1:** Comparison of T lymphocyte subsets and NK cells (mean ± SEM).

groups	CD8^+^ (%)	CD4^+^ (%)	NK (%)
patients in IT stage	27.06±6.14	38.59±14.48	10.15±8.52
patients in IA stage	24.17±6.69	44.03±10.38	6.93±4.82*
HI subjects	26.96±5.47	44.10±4.57	11.56±6.85

Compared to HI,

* p<0.05.

### IFN-γ and TNF-α expression in NK cells

**Figure 1:**
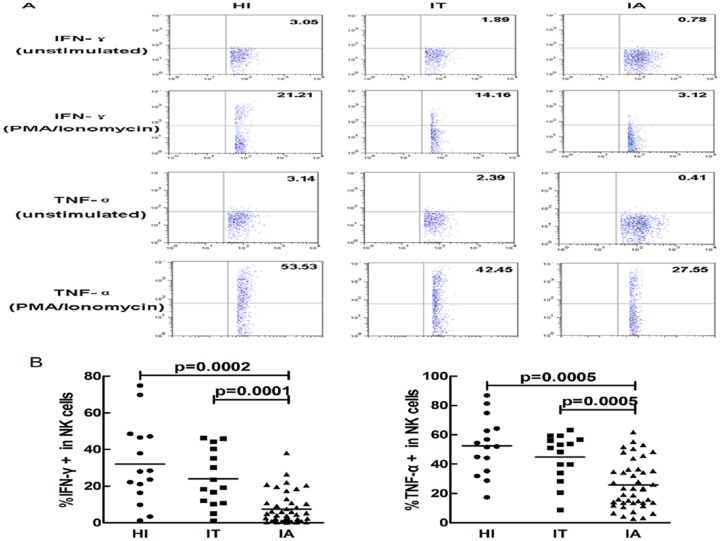
IFN-γ and TNF-α expression in peripheral NK cells. (A) The representative flow plots show IFN-γ and TNF-α expression including unstimulated controls and PMA/ionomycin stimulaton from the HI, IT and IA groups. (B) The percentage of peripheral NK cells expressing IFN-γ and TNF-α after stimulation with PMA/ionomycin in healthy individuals (HI) (circles), patients in the immune tolerant (IT) stage of CHB (squares) and patients in the immune activated (IA) stage of CHB (triangles) is shown. Each dot represents one individual. Horizontal lines represent the mean. The significance is calculated by the Mann-Whitney U test.

IFN-γ and TNF-α are pivotal functional cytokines for viral control and pathogenesis of hepatitis. After stimulated with PMA and ionomycin, IFN-γ and TNF-α expression in NK cells were detected. We found that NK cells from subjects with chronic HBV infection had a reduced capacity to produce IFN-γ and TNF-α. As shown in Fig. 1, expression of IFN-γ as well as TNF-α was remarkable down regulated in NK cells from patients with CHB in the IA stage as compared to patients in the IT stage and HI, respectively (p<0.05).

### NK cells cytotoxic activity

**Figure 2:**
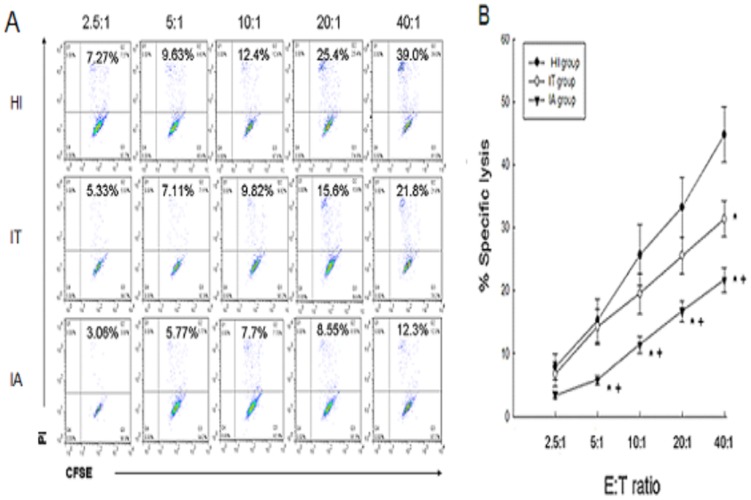
Cytotoxicity of peripheral NK cells. (A) Representative dot plots show K562 lysis by PBMCs from the three groups at different E∶T ratios (2.5∶1,5∶1,10∶1,20∶1, and 40∶1). (B) The cytotoxic activity of peripheral NK cells from the HI (n = 18), patients in the IT stage of CHB (n = 15), and patients in the IA stage of CHB (n = 42) at various E∶T ratios. The mean and standard error are shown. Asterisk represents p<0.05 compared to HI. Crosses represent p<0.05 compared to patients in the IT stage of CHB.

NK cells cytotoxicity was assessed by the analysis of different E∶T ratios. The cytotoxic activity of NK cells gradually increased with increasing E∶T ratios in the three study groups (Fig. 2A). As compared to HI, the cytotoxic activity of NK cells in patients with CHB was lower, especially in patients in the IA stage (p<0.05) (Fig. 2B).

### Expression of NK cells surface receptors

**Figure 3:**
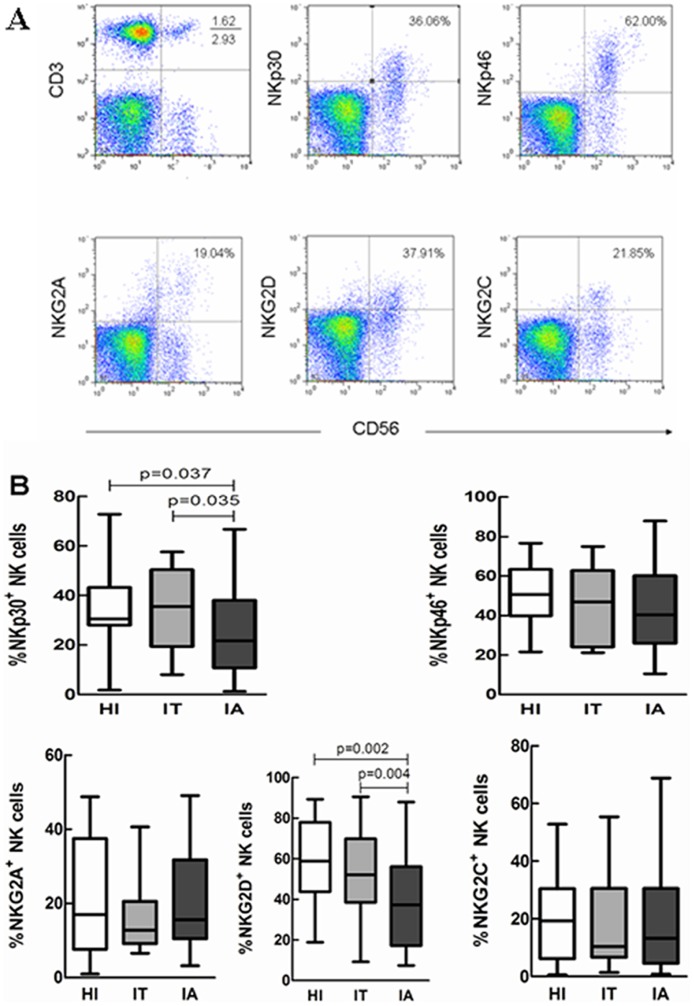
NK cell surface receptors expression in PBMC. (A) Representative dot plots of NK cells (CD3^−^CD56^+^) and their surface receptors (NKp30, NKp46, NKG2A, NKG2C, NKG2D) in PBMCs isolated from HBV-infected patients and HC are shown. (B) The percentage of NK cells expressing surface receptors in HI (n = 18), patients in the IT stage of CHB (n = 15), and patients in the IA stage of CHB (n = 42) is shown. The data are presented as boxplots showing medians (horizontal lines), upper and lower quartiles (boxes) and extreme values (whiskers). The significance is calculated by the Mann-Whitney U test.

We further analyzed NK cells receptors expression in the three study groups by flow cytometry (Fig. 3A). The receptors included the activating receptors NKp30, NKp46, NKG2D and NKG2C and the inhibitory receptor NKG2A. As illustrated in Fig. 3B, in patients with CHB in the IA stage the expression of the activating receptors NKp30 and NKG2D was down-regulated in peripheral NK cells compared to the patients in the IT stage and HI, respectively (p<0.05). No obvious difference of the expression of NKp46, NKG2C and NKG2A was observed in subjects in the three study groups.

### Correlation of peripheral NK cells activity with serum HBV DNA or ALT level

**Figure 4:**
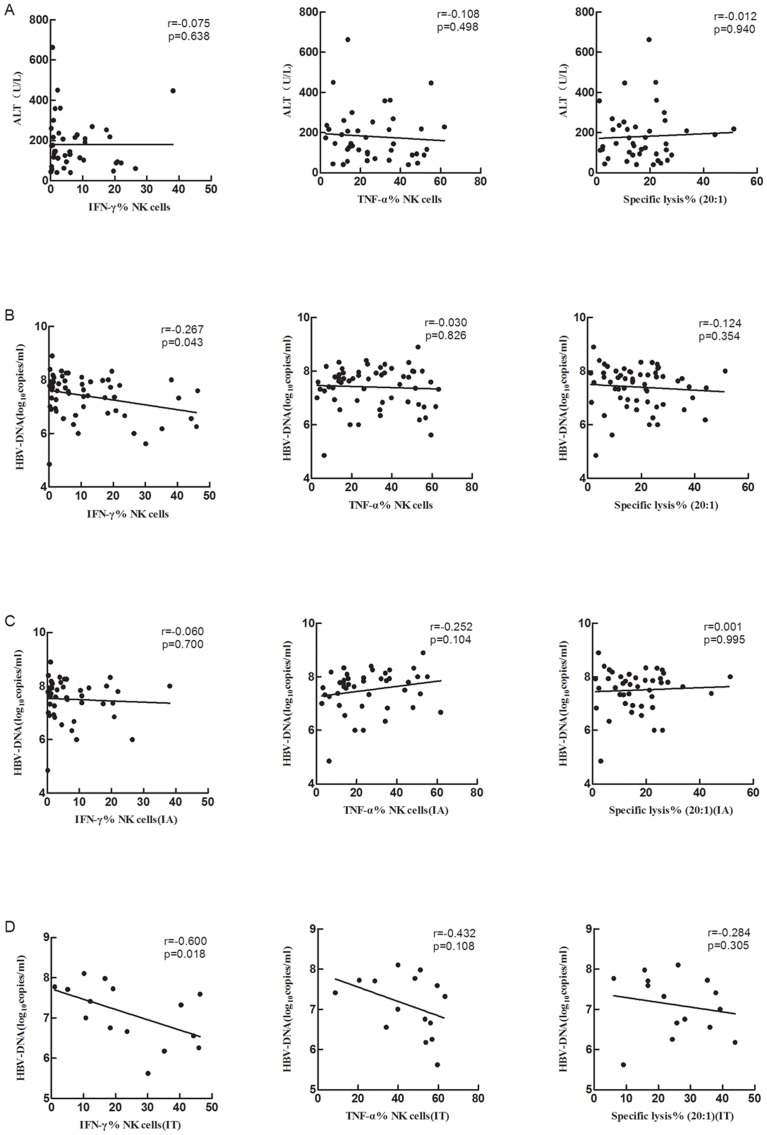
Relationship of NK cell activity with liver damage and HBV replication. Correlation between NK cells activity and ALT level in patients in the IA stage of CHB (A) and HBV replication in patients with CHB (B). Relationship of NK cell activity with HBV loads was further analyzed in IA stage (C) and in IT stage (D).

NK cells exert their function through cytotoxicity and secretion of cytokines. The relationship of NK cells activity with HBV replication and liver damage was analyzed in the study. As shown in Fig. 4, in patients with CHB in the IA stage, there was no correlation between NK cell activity and the ALT level (Fig. 4A). The level of IFN-γ but not of TNF-α expressed by NK cells was inversely correlated to serum HBV load in subjects with chronic HBV infection (Fig. 4B). The relationship between IFN-γ and serum HBV load was seen only in IT stage when patients were grouped (Fig. 4C, 4D). No correlation was observed between NK cells cytotoxicity and serum HBV load.

## Discussion

HBV infection can be roughly categorized in two stages: the IT and the IA stage, respectively, based on the serum HBV DNA level, serum ALT activity and the extent of liver damage. It is generally accepted that the HBV-specific immune response in the IT stage is lower than in the IA stage. Details regarding the innate immunity in the different stages of HBV infection are limited. NK cells,a major component of innate immune system, play a crucial role in the early clearance of HBV infection and the late developed adaptive immune response in CHB [32]. In this study we found that compared to health individuals, the frequency of peripheral NK cells was lower in patients with CHB in the IA stage. More important we demonstrated that the activity of NK cells, including cytotoxicity as well as IFN-γ and TNF-α production, was impaired in patients with CHB, especially in patients in the IA stage, and this functional impairment was companied with the phenotype changes of NK cells. Considering its significant role in anti-HBV infection, the reduced secretion of IFN-γ and TNF-α by NK cells, along with the decrease of its cytotoxicity which was shown in our study, might be associated with the chronicity of HBV infection.

It has been reported that the function of NK cells was dichotomy in chronic HBV and HCV infection [23], [26], which featuring in the conserved or enhanced cytolytic activity with the decreased cytokine production from NK cells. Zhang et al [24] also reported that NK cells from chronic hepatitis B patients had enhanced cytotoxicity but without the decrease of cytokine production. In contrast to these recent reports, our study shown that both the cytokine secretion and the cytolytic activity of NK cells were decreased in CHB patients, especially in patients in IA stage. The reason for this discrepancy is unknown, maybe partially due to the stimulation methods and target cells using in the cytotoxicity assay [33]. Indeed, as shown in the report from Oliviero and colleagues, the cytolytic activity of NK cells from CHB patients was significantly decreased when K562 cells was used as target cells while the cytotoxicity maintained when P815 target cells was used [23]. In our study, we also used K562 cells as the target cells for cytolytic assay. In this sense, our study had confirmed the results from Oliviero et al. Meanwhile, it was noteworthy to mention that there were also vigorous discrepancy reports of NK cells cytolytic activity in patients with chronic HCV infection [34]–[36]. The cytotoxicity of NK cells in CHB and CHC patients is thus needed to be further clarified. Methods used for stimulation of cytokine secretion by NK cells also might bring some bias on the results. Though PMA/ionomycin-stimulation is more potent, it is not as physiological as cytokines such as IL 12 and IL 18 in NK cells activation. However, the trends for the change of cytokines secretion by NK cells were similar when NK cells were stimulated with different activators [9].

NK cells activity is thought to be maintained by the integrated signals from the inhibitory and activating receptors of NK cells. The functional impairrment might be due to the phenotype change of NK cells. In our study, NK cells from patients with CHB in the IA stage expressed significantly lower levels of activating receptors NKp30 and NKG2D compared to patients in the IT stage and HI, respectively (p<0.05). Decreased expression of NK cells activating receptors was observed in the study from Tjwa et al [26] but not from Zhang et al [24]. In previous reports, however, some studies shown that the phenotype of NK cells was not accordant with the functional change of NK cells [23], [24], [26]. In our study, we found that the reduced NKp30 and NKG2D expression of NK cells was well consistent with a decrease of cytotoxicity and cytokines production of NK cells in patients with CHB compared to HI.

Recently, Zhang et al [24] had investigated the activity of NK cells from patients with chronic hepatitis B in great detail. They found that NK cells from the IA stage CHB patients were phenotypic and functional activated which correlated with liver damage. In contrast, our study shown that NK cells from CHB patients was immunological tolerance both in function and in phenotype. Currently, it is hard to explain this obvious difference. The differences of patient's age, viral loads and ALT level might account to some extent for the discrepancy. All the IA patients in our study were e antigen positive. This is quite important because, compared to those IA patients with e negative, e antigen positive may indicate a relative earlier stage in immune activation. In view of the quite long process of the immune activation in CHB patients, it is understandable that the immune response including NK cells reaction might be quite different or maybe even opposite from the beginning to the end of the immune activation in chronic HBV infection. The longitudinal compared study of NK cells function in CHB patients from the beginning of the infection to viral clearance is needed to clarify this very important issue. Indeed, different levels of NK cell activity as well as HBV-specific CD4 and CD8 T cell reaction from the incubation to the clinical acute and the convalescent phase in self-limited acute HBV infection had been reported by Dunn et al [37] recently.

It is interesting to note that NK cells activity is much lower in patients in IA stage as compared to the IT stage. This phenomenon might be a reflection of the time required to mount an effective adaptive antiviral immune response. In the early stage of HBV infection, the first-line of defense is the relative strong innate immune response, including NK cells activity. With the development of adaptive immunity, the innate NK cells function is reduced. In line with this hypothesis, Sprengers et al found that the number of intrahepatic NK cells in IT stage was higher than that in IA stage of patients with CHB, while HBV-specific T cells increased in IA stage along with the decrease of viral loads [5]. The temporal relationship of NK cells activity with viral- specific adaptive immune reaction was demonstrated also in HIV infection [38]. Alternatively, the reduced NK cells activity in patients in the IA stage might be one of the mechanisms resulting in viral immune escape.

While NK cells activity was found to correlate with viral clearance in acute self-limited HBV infection in the chimpanzee model, in patients the correlation between NK cells activity and the level of HBV replication and liver damage is still controversial [24], [26], [39]. In this study, we found that NK cells expressed IFN-γ level, but not TNF-α level and NK cells cytotoxicity, was inversely correlated with HBV replication in CHB patients. Our study confirmed the important role of IFN-γ in HBV inhibition. Interestingly, the inverse relationship was observed only in the IT stage but not in the IA stage patients. However, we did not found positive relationship between NK cells activity and liver damage (as indicated by serum ALT level) in the IA stage patients. In this context it is not clear whether peripheral NK cells activity reflects intrahepatic NK cells activity. Nevertheless, it is reasonable to assume that intrahepatic NK cell activity might correlate to liver damage. One can further assume that in patients with CHB the liver microenvironment has an important effect on immune effector cells, including NK cells, and thereby on HBV replication and the extent of liver damage.

In conclusion, our study shown that NK cells, at least in this group of patients with chronic hepatitis B was immune tolerant, especially in patients at IA stage, which characterized by the reduced cytotoxicity, the down regulated expression of IFN-γ and TNF-α, along with the decreased expression of activating receptors of NKp30 and NKG2D. This immune tolerant status of NK cells might take important role in the pathogenesis of chronic HBV infection.

## Materials and Methods

### Ethics Statement

All subjects signed an informed consent form to participate in the study. Our study obtained approval from the ethics committee of Tongji Hospital, Tongji Medical College, Huazhong University of Science and Technology. We obtain informed consent from the next of kin, caretakers, or guardians on the behalf of the minors/children participants involved in our study. The consent was written.

### Study Subjects

Overall, 57 patients with chronic HBV infection were recruited for the study and categorized according to the established criteria for viral hepatitis [28]–[29]. The immune tolerant (IT) stage was defined by high level HBV DNA in serum, HBeAg positivity and normal ALT levels. By contrast, the immune activated (IA) stage was defined by low level HBV DNA in serum and elevated ALT levels. All patients were negative for antibodies to hepatitis C virus, hepatitis D virus, human immunodeficiency virus, hepatitis A virus and hepatitis E virus. Further, the patients were not receiving immunosuppressive drugs during the last 6 months before recruitment and did not consume alcohol. Eighteen age and sex matched healthy individuals (HI) were enrolled as control group (Table 2). All subjects signed an informed consent form to participate in the study.

**Table 2:** Characteristics of study subjects.

	HI subjects (n = 18)	patients in IT stage (n = 15)	patients in IA stage (n = 42)
Age, yrs (mean±SEM)	25.35±9.65	28.27±8.93	30.82±10.58
Gender (Female∶male)	10∶8	8∶7	8∶34
ALT IU/L (mean±SEM)	20.50±1.50	22.20±7.12	199.85±192.93
HBV DNA log_10_ copies/ml (mean±SEM)	na	7.58±0.21	6.62±0.18

na = not applicable.

### Serological test of liver function, HBV markers and HBV DNA

ALT activity in serum was measured with routine automated techniques (upper limit: 40 U/L). The HBV markers HBsAg, anti-HBs, HBeAg, anti-HBe, anti-HBc total, and anti-HBc IgM were determined by commercially available enzyme-linked immunosorbent assays. Serum HBV DNA was quantitated by real-time PCR (RT-PCR) using a commercially available kit (Shanghai KH Biology Co. Ltd.) and the Lightcycler PCR system FQD-33A, Bioer. The lower detection limit was 500 viral genome copies/ml.

### Detection of lymphocyte subsets and NK cells surface receptors

Peripheral blood monouclear cells (PBMCs) were isolated from patients and healthy individuals, suspended in RPMI 1640 medium containing 2 mM/L L-glutamine and 10% fetal calf serum (Hyclone). Isolated PBMCs were stained with fluorochrome-conjugated antibodies to CD3-PerCP/cy5.5, CD56-FITC, CD8-FITC and CD4-FITC (Biolegend, San Diego, CA). NK cells surface receptors were stained with fluorochrome-conjugated antibodies to NKp46-PE, NKG2A-PE, NKp30-APC, NKG2C-APC, NKG2D-APC and isotype matched controls (Biolegend, San Diego, CA). Stained PBMC were isolated using a FACS Calibur flow cytometer (Becton Dickinson, USA) and analyzed by FlowJo analysis software (Treestar, Ashland, OR).

### Intracellular cytokine staining

After stimulation with 20 ng/ml phorbol myristate acetate (PMA) (Sigma, USA) plus 1 µg/ml ionomycin (Sigma) for 1 hour at 37°C in 5% CO_2_, cells were cultured for 4 hours in the presence of 2 µM/L monensin (Sigma). Then, cells were stained with fluorochrome-conjugated antibodies to CD3-PerCP/cy5.5, CD56-FITC, CD8-FITC and CD4-FITC (Biolegend, San Diego, CA) at 4°C in the dark. Cells were then fixed and permeabilized, followed by intracellular staining for IFN-γ-PE, TNF-α-PE and isotype matched controls (Biolegend, San Diego, CA) at 4°C in the dark. Finally, cells were detected by flow cytometry.

### Cytotoxicity assay

K562 cells were labeled with 3 µM/L carboxyfluorescein diacetate succinimidyl ester (CFSE) (Molecular Probes, Eugene, OR). PBMCs were subsequently incubated with the CFSE-labeled K562 cells at effector to target (E∶T) ratios of 2.5∶1, 5∶1, 10∶1, 20∶1, and 40∶1 and incubated for the 4 hours at 37°C in 5% CO_2_. Then, 100 µg/mL propidium iodide (PI) (BD Bioscience, San Jose, CA) was added to identify dead cells. The target cells alone were used as controls. The percentage lysis was calculated as: [(experimental release (%)−spontaneous release (%))/(1−spontaneous release (%))]×100.

### Statistical analysis

All data were analyzed with SPSS 13.0 for Windows (SPSS, Inc., Chicago, IL). Comparisons between various individuals were performed with the Mann-Whitney U test. Correlations between variables were calculated with the Spearman rank correlation test. P values<0.05 were considered significant.
